# Incorporating external evidence on between‐trial heterogeneity in network meta‐analysis

**DOI:** 10.1002/sim.8044

**Published:** 2018-11-28

**Authors:** Rebecca M. Turner, Clara P. Domínguez‐Islas, Dan Jackson, Kirsty M. Rhodes, Ian R. White

**Affiliations:** ^1^ MRC Clinical Trials Unit at UCL Institute of Clinical Trials and Methodology London UK; ^2^ MRC Biostatistics Unit Cambridge Institute of Public Health Cambridge UK; ^3^ Fred Hutchinson Cancer Research Center Seattle Washington; ^4^ Statistical Innovation Group, Advanced Analytics Centre AstraZeneca Cambridge UK

**Keywords:** Bayesian methods, heterogeneity, multiple‐treatments meta‐analysis, network meta‐analysis, prior distributions

## Abstract

In a network meta‐analysis, between‐study heterogeneity variances are often very imprecisely estimated because data are sparse, so standard errors of treatment differences can be highly unstable. External evidence can provide informative prior distributions for heterogeneity and, hence, improve inferences. We explore approaches for specifying informative priors for multiple heterogeneity variances in a network meta‐analysis.

First, we assume equal heterogeneity variances across all pairwise intervention comparisons (approach 1); incorporating an informative prior for the common variance is then straightforward. Models allowing unequal heterogeneity variances are more realistic; however, care must be taken to ensure implied variance‐covariance matrices remain valid. We consider three strategies for specifying informative priors for multiple unequal heterogeneity variances. Initially, we choose different informative priors according to intervention comparison type and assume heterogeneity to be proportional across comparison types and equal within comparison type (approach 2). Next, we allow all heterogeneity variances in the network to differ, while specifying a common informative prior for each. We explore two different approaches to this: placing priors on variances and correlations separately (approach 3) or using an informative inverse Wishart distribution (approach 4).

Our methods are exemplified through application to two network metaanalyses. Appropriate informative priors are obtained from previously published evidence‐based distributions for heterogeneity.

Relevant prior information on between‐study heterogeneity can be incorporated into network meta‐analyses, without needing to assume equal heterogeneity across treatment comparisons. The approaches proposed will be beneficial in sparse data sets and provide more appropriate intervals for treatment differences than those based on imprecise heterogeneity estimates.

## 1 INTRODUCTION

In a network meta‐analysis, the results from studies evaluating multiple different treatment comparisons are modelled simultaneously, and summary findings for each comparison are based on a combination of direct and indirect evidence. Network meta‐analysis enables estimation of treatment differences for which no direct evidence is available and may provide increased precision where little direct evidence is available. In addition, a network meta‐analysis allows calculation of probabilities that each treatment is *best* with respect to effectiveness or safety. When clinicians or health policy makers need to choose between several available treatments, findings from a network meta‐analysis are considerably more useful than the findings from a collection of separate pairwise meta‐analyses. Network meta‐analyses are increasingly widely reported in systematic reviews of health care interventions.^1^

It is common to assume that underlying treatment differences corresponding to each pairwise comparison are similar amongst the studies included in a network meta‐analysis, rather than identical. Between‐study heterogeneity may arise from differing study populations, differences in the conduct of the research, and biases caused by methodological flaws. A random‐effects network meta‐analysis model is often considered appropriate for allowing for this variability and leads to estimation of average treatment differences and between‐study heterogeneity variances for each pairwise comparison. The heterogeneity variances corresponding to multiple treatment comparisons may be assumed equal or unequal across comparisons. An assumption of equal heterogeneity variances simplifies the model and is commonly used but will not always be realistic. Networks may include several treatments of the same type (for example, various forms of radiotherapy (RT) or similar pharmacological treatments), in addition to treatments of a different type (for example, surgery) and a control treatment. Between‐study heterogeneity within a comparison of two pharmacological treatments, for example, might be expected to be lower than that within a pharmacological vs surgery comparison or a pharmacological vs control comparison.^2^ Lu and Ades have proposed models that allow heterogeneity variances to be unequal across treatment comparisons.^3^

Heterogeneity variances are often imprecisely estimated in pairwise meta‐analyses because many meta‐analyses in health research contain only a small number of studies.^4^ In a review of network meta‐analyses, the median number of studies per network was found to be 21 (inter‐quartile range 13 to 40) and the median number of studies per comparison was 2 (inter‐quartile range 1 to 4).^5^ Heterogeneity variances corresponding to individual treatment comparisons are therefore extremely likely to be imprecisely estimated. If heterogeneity is assumed equal across comparisons, increased precision is available for estimating the common heterogeneity variance but remains low in the many network meta‐analyses including few studies in total. Standard errors and confidence intervals for treatment differences depend directly on estimated heterogeneity variances, so imprecisely estimated heterogeneity could lead to misleading results and conclusions. When decisions are informed by predictive distributions for treatment effects, which allow for heterogeneity, an imprecise overestimate could exaggerate the uncertainty in the decision.

In previous work, we have presented predictive distributions for heterogeneity in a range of settings, constructed from meta‐analyses within the Cochrane Database of Systematic Reviews.^2^, ^6^, ^7^ When performing meta‐analysis within a Bayesian framework, we can specify such predictive distributions as informative prior distributions for heterogeneity and potentially gain in precision. In network meta‐analyses, estimation of heterogeneity for individual treatment comparisons is often even more difficult than in pairwise meta‐analyses. The aim of this paper is to explore the use of informative priors for the multiple heterogeneity variances corresponding to different treatment comparisons in a network meta‐analysis. We will use predictive distributions obtained from pairwise meta‐analyses because we have no reason to believe that between‐study heterogeneity would be different for treatment comparisons included in a network.

The layout of the paper is as follows. The basic structure of the random‐effects network meta‐analysis model is described in Section 2. In Section 3, we present four different approaches to specifying informative priors for multiple heterogeneity variances. In Section 4, the methods are applied to two example data sets. We conclude with a discussion in Section 5.

## 2 NETWORK META‐ANALYSIS MODEL

We assume a random‐effects model for pairwise comparisons of multiple treatments, as proposed initially by Higgins and Whitehead,^8^ and later extended by Lu and Ades.^9^ We use a contrast‐based approach to modelling between‐study variability, in which assumptions of exchangeability are made for pairwise treatment differences rather than for treatment arms, as in conventional pairwise meta‐analysis models.^10^ We focus on models for binary outcome data. All models are fitted within a Bayesian framework.

In a random‐effects network meta‐analysis model, we denote the underlying treatment difference in study *j* by *δ*_*jXY*_, for comparison of treatment Y vs treatment X. For each comparison, treatment differences are assumed exchangeable across studies and drawn from a normal distribution, with mean *d*_*XY*_ and between‐study heterogeneity variance $\tau_{\mathrm{XY}}^{2}$. In each study, one treatment is regarded as the baseline treatment, against which treatment differences are defined. For the binomial data in study *j* under treatment *k*, the full model is

(1) $$\begin{array}{cc} r_{\mathrm{jk}}∼ & \text{Bin}\;\left(\pi_{\mathrm{jk}},n_{\mathrm{jk}}\right)\\ \text{logit}\;\left(\pi_{\mathrm{jk}}\right)= & \left\{\begin{array}{c} \mu_{j},\;k=b_{j}\\ \mu_{j}+\delta_{\mathrm{jbk}},\;k>b_{j}, \end{array}\right. \end{array}$$

where *b*_*j*_ (written as *b* in subscripts, for clarity) indicates the baseline treatment in study *j*. The *μ*_*j*_ represent the baseline log odds in each study, assumed unrelated to each other and treated as fixed effects. We choose vague Normal(0, 10^4^) priors for the *μ*_*j*_. The assumptions made about the random effects *δ*_*jbk*_ are described below.

Throughout this paper, we assume consistency for the evidence available from a network meta‐analysis, meaning that we assume agreement between the direct and indirect evidence informing each treatment comparison.^11^ Suppose the network includes a total of *p* + 1 treatments, where treatment 0 is regarded as the overall reference treatment (usually representing control or standard care). Following Lu and Ades,^11^ the contrasts with the reference treatment, *d*_01_,*d*_02_, …,*d*_0*p*_, are referred to as the *basic parameters* in model (1). Under the assumption of consistency, the remaining treatment contrasts are referred to as *functional parameters* that can be expressed in terms of the basic parameters, for example, *d*_12_ = *d*_02_ − *d*_01_, *d*_13_ = *d*_03_ − *d*_01_, and so on. These relationships are referred to as the consistency equations. We choose vague Normal(0, 10^4^) priors for the basic parameters *d*_01_,*d*_02_, …,*d*_0*p*_.

We now write the random part of model (1) in vector form as follows:

$$\delta_{j}∼N\;\left(d,\Sigma \right),$$

where **δ**_*j*_ = (*δ*_*j*01_, *δ*_*j*02_, …, *δ*_*j*0*p*_) is the vector of contrasts with the reference treatment in study *j* and ***d*** is the vector of basic parameters. The diagonal entries Σ_*kk*_ of **Σ** are the heterogeneity variances $\tau_{01}^{2},\tau_{02}^{2},\text{…},\tau_{0p}^{2}$ corresponding to contrasts with the reference treatment. Heterogeneity variances corresponding to contrasts not involving the reference treatment are given by $\tau_{\mathrm{kl}}^{2}=\text{Var}\left(\delta_{j0k}-\delta_{j0l}\right)=\Sigma_{\mathrm{kk}}+\Sigma_{\mathrm{ll}}-2\Sigma_{\mathrm{kl}}$. When choosing priors for **Σ**, it is important to ensure sensible relationships amongst the heterogeneity variances. Lu and Ades show that *second‐order consistency* should hold for the heterogeneity variances relating to the three treatment contrasts amongst any treatment triple {A,B,C}.^3^ We will ensure this, for any network size, by requiring that **Σ** is positive semidefinite. We note that this requirement is more conceptual than technical. Models which do not ensure second‐order consistency can be fitted and estimation can be achieved without any computational problems, but the validity of the resulting inference would be questionable and the estimates would be difficult to interpret. Therefore, we believe it is better to fit models which are conceptually plausible.

## 3 SPECIFYING INFORMATIVE PRIORS FOR BETWEEN‐STUDY HETEROGENEITY

We will consider several alternative strategies for specifying informative priors for multiple heterogeneity variances in a network meta‐analysis. The assumptions made under these strategies are summarised in Table 1.

**Table 1:** Assumptions made under approaches 1 to 4, and strategies for matching parameters to available data‐based priors for heterogeneity

Approach	Structural	Prior Distribution(s)	Parameters of Prior	What to Match	Target for Match	Ensures Σ Is
	Assumptions					Positive semidefinite?
1	$\tau_{\mathrm{kl}}^{2}=\tau^{2}$	log(*τ*^2^)∼*N*(*m*, *s*^2^)	(*m*, *s*^2^)	Mean and variance	Data‐based priors	Yes
				of log(*τ*^2^)	for $\tau_{\mathrm{kl}}^{2}$, reported by	
					outcome type (Table 2)	
2	$\tau_{\mathrm{kl}}^{2}=\tau^{2}\text{exp}\left(m_{\mathrm{kl}}\right)$	log(*τ*^2^)∼*N*(0, *s*^2^)	(*m*_*kl*_, *s*^2^)	Mean and variance	Data‐based priors	Depends on nature of
				of $\log\left(\tau_{\mathrm{kl}}^{2}\right)$	for $\tau_{\mathrm{kl}}^{2}$, reported by	treatments
					outcome and	
					comparison type (Table S1)	
3	$\tau_{\mathrm{kl}}^{2}=\tau_{k}^{2}+\tau_{l}^{2}-2\rho_{\mathrm{kl}}\tau_{k}\tau_{l}$	$\text{log}\left(\tau_{k}^{2}\right)∼N\left(m,s^{2}\right)$	(*m*, *s*^2^, *a*, *b*)	Mean and variance	Data‐based priors	Yes
	*ρ*_*kl*_∼*f*(***ϕ***)	cos(*ϕ*_*kl*_)∼Beta(*a*, *b*)		of $\log\left(\tau_{k}^{2}\right)$; Beta	for $\tau_{k}^{2}$, reported by	
				parameters *a*,*b*	outcome type (Table 2); Beta	
					prior chosen according to	
					network size (Table 3)	
4	None	$\Sigma^{-1}=\frac{M}{\delta }$	$\left(S,t,m_{\delta },s_{\delta }^{2}\right)$	Mean and variance	Data‐based priors	Yes
		***M***∼Wishart(***S***, *t*)	*t* = *p* + 1	of log(*δ*), matched	for $\tau_{\mathrm{kl}}^{2}$, reported by	
		$\log\left(\delta \right)∼N\left(m_{\delta },s_{\delta }^{2}\right)$		using (6)	outcome type (Table 2)	

### 3.1 Equal heterogeneity variances (approach 1)

The simplest approach to ensuring second‐order consistency is to assume that heterogeneity variances $\tau_{\mathrm{kl}}^{2}$ for all treatment comparisons in the network are equal. Under this assumption, Σ = *τ*^2^**P**, where **P** is the *p*×*p* matrix with 1's on the diagonal and 0.5's off the diagonal, so Σ is guaranteed to be positive semidefinite. We can then choose a single informative prior for the common heterogeneity variance *τ*^2^.

When assuming equal heterogeneity variances, we assume log(*τ*^2^)∼*N*(*m*, *s*^2^), where choice of the mean *m* and variance *s*^2^ for log heterogeneity is based on external data.^2^, ^7^ In previous work, we have presented data‐based log‐Normal distributions as informative prior distributions for between‐study heterogeneity in binary outcome meta‐analyses, for a variety of settings defined by outcome type and intervention comparison type.^2^, ^7^ If there are several different types of intervention comparison in the network, it might be reasonable to choose the prior that best matches the majority of intervention comparisons or to choose the widest of the priors as the common prior, as a conservative approach.

Although practical, the assumption that all heterogeneity variances are equal is a strong assumption, which we would like to relax, using the models discussed in the following sections. However, we note that this assumption may be considered plausible in some networks.

### 3.2 Proportional heterogeneity variances with different informative priors (approach 2)

We now consider how we could specify different informative priors for some heterogeneity variances within a network. Informed by previous empirical evidence,^7^ we would like to select priors according to whether the treatments compared were both active, or active and placebo/control, and whether the active treatments were pharmacological or nonpharmacological. This categorisation of intervention comparison types leads to five different possible priors, with different means and variances, for each contrast within a meta‐analysis (Table S1).

The simplest way to allow different informative priors across the network is to assume the heterogeneity variances to be proportional rather than equal. We consider allowing the prior means *m*_*kl*_ (on the log heterogeneity scale) to be unequal across treatment comparisons, while still assuming equality for the prior variances, as follows:

(2) $$\tau^{2}∼\text{log}‐N\;\left(0,s^{2}\right)\;\text{and}\;\tau_{\mathrm{kl}}^{2}=\tau^{2}\text{exp}\left(m_{\mathrm{kl}}\right)\forall k,l.$$

Under (2), the variances for the $\text{log}\left(\tau_{\mathrm{kl}}^{2}\right)$ are equal to *s*^2^ for all *k*, *l*, whereas the means *m*_*kl*_ differ.

In Supplementary Appendix A1, we explore the conditions under which separate priors for $\tau_{\mathrm{kl}}^{2}$ of the form (2) result in positive semidefiniteness for **Σ**, for the specific priors presented in Table S1. We note that this investigation would need to be repeated if a different set of data‐based priors was used. We have considered networks including up to *p* = 100 active treatments, although higher values in this range are very unlikely to occur in clinical research. For up to *p* = 4 active treatments, we find that **Σ** is positive semidefinite for all network types. For larger networks, positive semidefiniteness holds for all networks in which all treatments are pharmacological or nonpharmacological. However, when the reference treatment is placebo/control and *p* > 4, positive semidefiniteness holds for only a minority of network types: when all active treatments are pharmacological, when all or all but one active treatments are nonpharmacological, or when all but two active treatments are nonpharmacological if *p* ≤ 88. For example, a simple network in which positive semidefiniteness does not hold is a network comparing four pharmacological treatments and one nonpharmacological treatment, with a placebo as reference treatment. In networks for which assuming proportional heterogeneity variances does not ensure positive semidefiniteness for **Σ**, we recommend using the approach described in Section 3.3.

### 3.3 Unequal heterogeneity variances with a common informative prior (approach 3)

We now explore how to specify informative priors for heterogeneity while allowing the heterogeneity variances $\tau_{\mathrm{kl}}^{2}$ to differ across treatment comparisons. Lu and Ades^3^ have previously proposed a model which allows unequal $\tau_{\mathrm{kl}}^{2}$ while ensuring that the covariance matrix **Σ** remains positive semidefinite, so that second‐order consistency holds. Under their approach, the marginal priors implied for the heterogeneity variances are not immediately apparent, and therefore, it is not straightforward to specify informative priors. We follow their approach and propose how to incorporate informative priors.

To ensure that appropriate constraints are met, Lu and Ades introduced some additional parameters $\tau_{k}^{2}$, defined by the following relationships:

(3) $$\tau_{\mathrm{kl}}^{2}=\tau_{k}^{2}+\tau_{l}^{2}-2\rho_{\mathrm{kl}}\tau_{k}\tau_{l},$$

where $\tau_{k}^{2}$ and $\tau_{l}^{2}$ are, respectively, regarded as variances of random quantities *θ*_*jk*_ and *θ*_*jl*_ that can be interpreted as the random effects of corresponding treatment arms *k* and *l* up to a common unknown constant.^3^ Care must be taken over choice of priors for the correlations *ρ*_*kl*_, to ensure a valid between‐arm correlation matrix **R**.

Following this approach, we need to place priors directly on the treatment arm‐specific variance parameters $\tau_{k}^{2}$. However, the data‐based predictive distributions that we plan to use as informative priors are available for variances corresponding to treatment *comparisons* rather than treatment *arms*, that is, for the original model (1) parameters $\tau_{\mathrm{kl}}^{2}$. We aim to choose suitable informative priors for the arm‐specific variances $\tau_{k}^{2}$, which will imply our target data‐based priors for the comparison‐specific variances $\tau_{\mathrm{kl}}^{2}$. We do this by considering the relationship between the priors specified for the $\tau_{k}^{2}$ and the *ρ*_*kl*_ in (3), and the resulting implied priors for the $\tau_{\mathrm{kl}}^{2}$.

We plan to specify a common data‐based log‐Normal prior for all $\tau_{\mathrm{kl}}^{2}$, for practical reasons; in the Discussion, we explain why it would be considerably more difficult to implement this approach if using multiple different informative priors. Because the target data‐based priors for the $\tau_{\mathrm{kl}}^{2}$ are log‐Normal, we also consider specifying log‐Normal priors for the $\tau_{k}^{2}$. To ensure a common prior for the $\tau_{\mathrm{kl}}^{2}$, we will choose identical priors for each $\tau_{k}^{2}$.

To find a suitable log‐Normal prior for the $\tau_{k}^{2}$, we propose matching the moments of the implied prior for $\tau_{\mathrm{kl}}^{2}$ based on expression (3) with the known moments of a chosen data‐based informative prior $\tau_{\mathrm{kl}}^{2}∼\text{log}‐N\left(m_{D},s_{D}^{2}\right)$. We assume log ‐ N(*m*, *s*^2^) priors for each of the $\tau_{k}^{2}$. Independently of $\tau_{k}^{2}$ and $\tau_{l}^{2}$, we assume a generic prior distribution for the correlation *ρ*_*kl*_ in (3), with mean *m*_*ρ*_ and variance $s_{\rho }^{2}$. By equating the mean and variance of the implied prior for $\tau_{\mathrm{kl}}^{2}$ with the mean and variance of the data‐based prior (details given in supplementary appendix A2), we obtain the following:

(4) $$\begin{array}{ll} \;E\;\left(\tau_{\mathrm{kl}}^{2}\right) & =e^{m_{D}}e^{s_{D}^{2}/2}=2e^{m}e^{s^{2}/4}\left(e^{s^{2}/4}-m_{\rho }\right)\\ \mathrm{Var}\;\left(\tau_{\mathrm{kl}}^{2}\right) & =e^{2m_{D}}e^{s_{D}^{2}}\left(e^{s_{D}^{2}}-1\right)=2e^{2m}e^{s^{2}}\left(e^{s^{2}}-1+2s_{\rho }^{2}\right)+4m_{\rho }^{2}e^{2m}e^{s^{2}/2}\left(e^{s^{2}/2}-1\right)\\ -\;8m_{\rho }e^{2m}e^{3s^{2}/4}\left(e^{s^{2}/2}+1\right).\; \end{array}$$

For *m*_*D*_ and *s*_*D*_ corresponding to a chosen data‐based prior for $\tau_{\mathrm{kl}}^{2}$ and given the mean and variance $\left\{m_{\rho },s_{\rho }^{2}\right\}$ of the prior specified for *ρ*_*kl*_, we can solve the above equations for *m* and *s*, using numerical methods (for example, using the package nleqslv in R^12^), and find solutions *m* = *m*_*A*_ and *s* = *s*_*A*_. We can now declare $\text{log}‐N\left(m_{A},s_{A}^{2}\right)$ priors for all $\tau_{k}^{2}$, in order that the implied priors for the $\tau_{\mathrm{kl}}^{2}$ will have the target data‐based mean *m*_*D*_ and variance $s_{D}^{2}$. However, the implied priors will not be log‐Normal and do not follow a known distribution.

For example, if our chosen data‐based prior for the $\tau_{\mathrm{kl}}^{2}$ is log‐N(−4.28, 1.61^2^) and we use a prior for *ρ*_*kl*_ with mean *m*_*ρ*_ = 0.5 and variance $s_{\rho }^{2}=0.07$, we solve (4) and find solutions *m* =  −4.83 and *s* = 1.69. We would therefore declare log‐N(−4.83, 1.69^2^) priors for all $\tau_{k}^{2}$, in order that the implied priors for the $\tau_{\mathrm{kl}}^{2}$ have the target data‐based mean and variance.

To provide suitable prior distributions for the correlation matrix **R** in Section 3.2.1, Lu and Ades used a Cholesky decomposition to write **R** = **L**^*T*^**L**, where **L** is an upper‐triangular matrix and, then, used a spherical parameterization.^13^ For example, in a network with three treatments,

(5) $$\begin{array}{ll} \rho_{12} & =\text{cos}\left(\phi_{12}\right)\\ \rho_{13} & =\text{cos}\left(\phi_{13}\right)\\ \rho_{23} & =\text{cos}\left(\phi_{12}\right)\text{cos}\left(\phi_{13}\right)+\text{sin}\left(\phi_{12}\right)\text{sin}\left(\phi_{13}\right)\text{cos}\left(\phi_{23}\right). \end{array}$$

In Supplementary Appendix A3, we provide details of the priors chosen for the cos(*ϕ*_*kl*_).

#### 3.3.1 Implementation

To assist with implementation of the approach described above, we will use (4) to find priors suitable for the arm‐specific variance parameters $\tau_{k}^{2}$, which correspond to a set of data‐based priors for the $\tau_{\mathrm{kl}}^{2}$. In approach 3, we allow the $\tau_{\mathrm{kl}}^{2}$ to vary across treatment comparisons but specify a common informative prior for all $\tau_{\mathrm{kl}}^{2}$. It would be convenient to have data‐based predictive distributions for $\tau_{\mathrm{kl}}^{2}$, which do not depend on comparison type, for use in approach 3 and in approach 1 where equal heterogeneity variances are assumed. We have therefore fitted a revised model to meta‐analyses from the Cochrane Database of Systematic Reviews, based on the models fitted by Turner et al,^7^ in which only outcome type is used as a predictor of between‐study heterogeneity. Table 2 presents the predictive distributions obtained from this model. These would be suitable as informative priors for networks including a mixture of treatment comparison types, but if the majority of comparisons were of the same type (eg, pharmacological vs pharmacological), it would be preferable to use the predictive distribution available for that particular comparison. Next, we used numerical methods to solve the equations in (4) for each predictive distribution for the $\tau_{\mathrm{kl}}^{2}$, to find distributions suitable as log‐normal priors for the $\tau_{k}^{2}$ in (3), which will imply the chosen data‐based priors.

**Table 2:** Data‐based predictive distributions for heterogeneity variances $\tau_{\mathrm{kl}}^{2}$, by outcome type, and corresponding distributions for the $\tau_{k}^{2}$

	Predictive Distribution for the $\tau_{kl}^{2}$	Corresponding Distribution for the $\tau_{k}^{2}$,
		Based on Matching Moments in (6), a
All‐cause mortality	LN(−4.28, 1.61^2^)	LN(−4.83, 1.69^2^)
Obstetric outcomes	LN(−3.33, 1.60^2^)	LN(−3.88, 1.69^2^)
Cause‐specific mortality/major morbidity	LN(−3.52, 1.61^2^)	LN(−4.08, 1.70^2^)
event/composite (mortality or morbidity)		
Resource use/hospital stay/process	LN(−2.21, 1.60^2^)	LN(−2.76, 1.69^2^)
Surgical/device‐related success/failure	LN(−1.86, 1.61^2^)	LN(−2.42, 1.70^2^)
Withdrawals/dropouts	LN(−2.85, 1.60^2^)	LN(−3.40, 1.69^2^)
Internal/external structure‐related outcomes	LN(−2.53, 1.61^2^)	LN(−3.09, 1.70^2^)
General physical health indicators	LN(−2.37, 1.61^2^)	LN(−2.93, 1.70^2^)
Adverse events	LN(−1.97, 1.60^2^)	LN(−2.52, 1.69^2^)
Infection/onset of new disease	LN(−2.55, 1.60^2^)	LN(−3.10, 1.69^2^)
Signs/symptoms reflecting continuation/	LN(−2.13, 1.60^2^)	LN(−2.68, 1.69^2^)
end of condition		
Pain	LN(−1.85, 1.60^2^)	LN(−2.40, 1.69^2^)
Quality of life/functioning (dichotomised)	LN(−2.59, 1.62^2^)	LN(−3.15, 1.71^2^)
Mental health indicators	LN(−2.20, 1.62^2^)	LN(−2.76, 1.71^2^)
Biological markers (dichotomised)	LN(−1.83, 1.60^2^)	LN(−2.38, 1.69^2^)
Subjective outcomes (various)	LN(−2.75, 1.61^2^)	LN(−3.31, 1.70^2^)

a Assuming that priors for the correlations *ρ*_*km*_ have mean 0.5 and variance 0.07, as when using the priors listed in Table 3.

In order to solve (4), we needed to assume values for the mean *μ*_*ρ*_ and variance $\sigma_{\rho }^{2}$ of the prior distributions for the correlations *ρ*_*kl*_. The *ρ*_*kl*_ represent correlations between sets of random effects for treatment arms *k* and *l*. Given that all *ρ*_*kl*_ are equal to 0.5 in the common heterogeneity variances model (approach 1) and assuming that the *ρ*_*kl*_ are very likely to be positive, we consider a Uniform(0, 1) distribution to be a suitable choice of prior. We have chosen to assume *m*_*ρ*_ = 0.5 and $s_{\rho }^{2}=0.07$ because these values approximate the mean and variance of a correlation coefficient under a Uniform(0, 1) distribution, conditional on positive semidefiniteness. We have identified Beta priors for the cos(*ϕ*_*kl*_) in (S4) (see Supplementary Appendix A3), which will imply priors for the *ρ*_*kl*_ with these moments, for given network sizes up to 10 (Table 3).

**Table 3:** Prior distributions for the cos(ϕ_kl_) in (S4), for use with the distributions given for the $\tau_{k}^{2}$ in Table 2

Number of Treatments in Network	Beta Prior for the cos(*ϕ*_*km*_)
4	Beta(0.93, 1.07)
5	Beta(0.82, 0.98)
6	Beta(0.81, 0.99)
7	Beta(0.71, 0.89)
8	Beta(0.71, 0.89)
9	Beta(0.62, 0.78)
10	Beta(0.62, 0.78)

### 3.4 Informative inverse Wishart priors (approach 4)

The inverse Wishart distribution is a common choice of prior distribution for a covariance matrix and ensures positive semidefiniteness.^14^ Here, we explore how to choose informative inverse Wishart prior distributions for which the marginal priors for the heterogeneity variances $\tau_{\mathrm{kl}}^{2}$ approximately match specified data‐based priors.

We first consider declaring an inverse Wishart distribution for **Σ** in model (1), of the form **Σ**^−1^∼Wishart(**S**, *t*), where **S** is a *p*×*p* matrix and *t* represents degrees of freedom. Heterogeneity variances representing contrasts with the reference treatment are the diagonal elements of **Σ**, say, Σ_*kk*_. The marginal distribution for each Σ_*kk*_ is an inverse gamma distribution: Σ_*kk*_∼*IG*((*t* − *p* + 1)/2, *S*_*kk*_/2).^15^ Selection of **S** and *t* can be informed by considering the moments of the implied priors for the log heterogeneity variances, log(Σ_*kk*_).

If we were to use a standard inverse Wishart distribution, **Σ**^−1^∼Wishart(**S**, *t*), the variance for log(Σ_*kk*_) would be fixed at *ψ*_1_((*t* − *p* + 1)/2) for all **S**, where *ψ*_1_ is the trigamma function, so it would not be possible to match the variance for log(Σ_*kk*_) to a chosen data‐based value. We therefore instead use a scaled inverse Wishart distribution. We use a scaling parameter *λ* and assume **Σ**^−1^∼**M**/*λ*, where **M**∼Wishart(**S**, *t*) and $\text{log}\left(\lambda \right)∼\text{Normal}\left(m_{\lambda },s_{\lambda }^{2}\right)$. By considering the resulting mean and variance for log(Σ_*kk*_), we find that we can match these to a target data‐based prior distribution, $\text{log}\left(\Sigma_{\mathrm{kk}}\right)∼N\left(m_{D},s_{D}^{2}\right)$, by setting

(6) $$m_{\lambda }=m_{D}-\text{log}\left(S_{\mathrm{kk}}/2\right)+\psi \left(\left(t-p+1\right)/2\right)\;\;s_{\lambda }^{2}=s_{D}^{2}-\psi_{1}\left(\left(t-p+1\right)/2\right),$$

where *ψ* is the digamma function.

We need to choose a value for the degrees of freedom *t,* which must be greater than or equal to the dimension *p*. Matching to the target variances $s_{D}^{2}$ in Table S1 would not be possible if *t = p*, since *ψ*_1_(1/2) is then greater than all values of $s_{D}^{2}$. As the degrees of freedom of the Wishart distribution increase, the prior correlations between the heterogeneity variances $\tau_{\mathrm{kl}}^{2}$ also increase. By assuming higher prior correlations between the $\tau_{\mathrm{kl}}^{2}$, we would increase the amount of borrowing across treatment comparisons. We choose to set *t* = *p* + 1, which leads to the lowest correlations and thus the lowest amount of borrowing across comparisons under this approach, while allowing matching to the target variances $s_{D}^{2}$.

The magnitude of *S*_*kk*_ is inconsequential and does not affect the mean of log(Σ_*kk*_) because we adjust for this value when choosing *m*_*λ*_; we set *S*_*kk*_ = 1. Heterogeneity variances representing contrasts between two nonreference treatments *k* and *l* are given by Σ_*kk*_ + Σ_*ll*_ − 2Σ_*kl*_. The value for *S*_*kl*_ is set to *S*_*kk*_/2 = 0.5 to ensure that the implied distributions for these heterogeneity variances are identical to those for heterogeneity variances representing contrasts with the reference treatment (see Supplementary Appendix A4).

## 4 APPLICATION TO EXAMPLE NETWORK META‐ANALYSES

### 4.1 Network of treatments for smoking cessation

**Figure 1:**
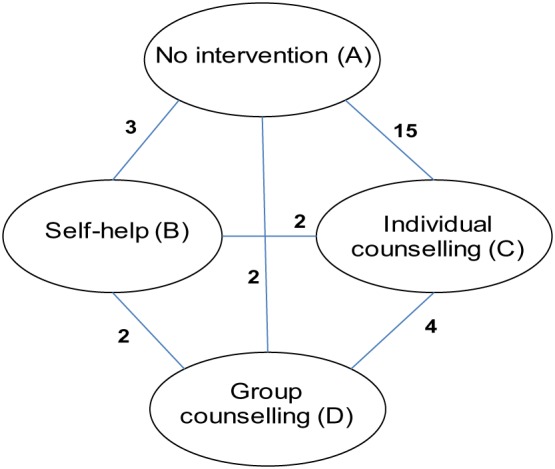
Network meta‐analysis comparing treatments for smoking cessation counselling [Colour figure can be viewed at wileyonlinelibrary.com]

To illustrate the above methods, we first reanalyse a commonly used network meta‐analysis data set including 24 trials comparing treatments for smoking cessation counselling: no intervention (A), self‐help (B), individual counselling (C), and group counselling (D) (Figure 1).^16^ The outcome reported is successful cessation of smoking at 6 to 12 months. Direct evidence is available on all six pairwise comparisons: AB (three trials), AC (15 trials), AD (two trials), BC (two trials), BD (two trials), CD (four trials). There are two three‐arm trials in the data set.

Initially, we assumed heterogeneity variances corresponding to all treatment comparisons in the network to be equal (approach 1). We first chose a vague Uniform(0, 2) prior for the common between‐study standard deviation *τ* (given that analyses are on the log odds ratio scale), and subsequently specified an informative log‐Normal(−2.75, 1.61^2^) prior for *τ*^2^. This distribution represents a data‐based predictive distribution for heterogeneity in a meta‐analysis examining subjective outcomes (Table 2). Under model (1), each three‐arm trial *j* produces two correlated random effects *δ*_*jbX*_ and *δ*_*jbY*_. We have allowed for this by assuming the second, *δ*_*jbY*_, to be randomly distributed conditional on the first, *δ*_*jbX*_, following the approach proposed by Dias et al.^17^

In approach 2, we first used vague priors by assuming $\tau_{0l}^{2}=\tau^{2}$ for all *l* and $\tau_{\mathrm{kl}}^{2}=\tau^{2}\text{exp}\left(m\right)$ for *k* ≠ 0, with *τ*∼Uniform(0, 2) and *m*∼*N*(0, 0.5^2^), thus assuming that heterogeneity is different (and proportional) for active vs control and active vs active comparisons. We then chose separate informative priors for heterogeneity variances corresponding to nonpharmacological vs nonpharmacological comparisons (log‐Normal(−2.26, 1.45^2^)) and nonpharmacological vs control comparisons (log‐Normal(−2.92, 1.71^2^)) (Table S1), which have medians of 0.32 and 0.23, respectively, where treatments B, C, and D are nonpharmacological and treatment A is a control treatment. As standard deviation of the prior for *τ*^2^ in (2), we used a weighted average of the standard deviations from the two predictive distributions, using the number of study comparisons of each type as weights.

We then allowed heterogeneity variances to vary across treatment comparisons (approach 3). We first declared vague Uniform(0, 2) priors for *τ*_0_, *τ*_1_, *τ*_2_, *τ*_3_ and, then, log‐Normal(−3.31, 1.70^2^) priors for $\tau_{0}^{2},\tau_{1}^{2},\tau_{2}^{2},\tau_{3}^{2}$, which imply priors with the same mean and variance as the chosen log‐Normal(−2.75, 1.61^2^) priors for the $\tau_{\mathrm{kl}}^{2}$ (Table 2). Next, we used a scaled inverse Wishart distribution with four degrees of freedom (approach 4), with the prior for the scaling parameter chosen to match the marginal priors for the $\tau_{\mathrm{kl}}^{2}$ to a log‐Normal(−2.75, 1.61^2^) distribution.

Approaches 1, 2, and 4 were implemented in WinBUGS^18^ and approach 3 was implemented in OpenBUGS^19^ (since the updating algorithm required was only available in OpenBUGS). We based results on 100 000 Markov chain Monte Carlo iterations, following a burn‐in period of 20 000 iterations, which was sufficient to achieve convergence. Code to implement approaches 1 to 4 is provided in Supplementary Appendix A5.

Using informative log‐Normal priors rather than vague priors in the equal and unequal variance models (approaches 1 and 3) has led to smaller heterogeneity estimates, with narrower credible intervals (Table 4). This has caused small changes to the central estimates (posterior medians) of the log odds ratios, and their 95% credible intervals have narrowed. The changes are greater in the unequal variances model (approach 3), in which few trials contributed to the estimation of between‐study heterogeneity variances corresponding to most comparisons (except AC). We note that the heterogeneity standard deviations for comparisons BC, BD, and CD are substantially smaller under the unequal variances model than under the equal variances model and close to the prior median of 0.25, and the corresponding log odds ratios for comparisons BC, BD, and CD (not shown) therefore have much narrower intervals than under the equal variances model. Although the heterogeneity variance for comparison AD is slightly higher under the unequal variances model, the contribution of more precise indirect evidence has caused the interval for the log odds ratio for AD to narrow. The heterogeneity variance for AC is estimated with most precision because this comparison has the largest amount of evidence (15 trials).

**Table 4:** Comparison of four treatments^a^ promoting smoking cessation when assuming equal, proportional, or unequal heterogeneity variances: posterior medians and 95% credible intervals for log odds ratios and between‐trial standard deviations, for the outcome of smoking cessation at 12 months

	Approach 1:	Approach 1:	Approach 2:	Approach 2:	Approach 3:	Approach 3:	Approach 4:
	Equal Variances	Equal Variances	Proportional	Proportional	Unequal Variances	Unequal Variances	Scaled Inverse
	(Vague Prior)	(Inf. Prior)	Variances (Vague Priors)	Variances (Inf. Priors)	(Vague Priors)	(Inf. Priors)	Wishart (Inf. Prior)
*d*_*AB*_	0.48 (−0.29, 1.30)	0.47 (−0.23, 1.20)	0.51 (−0.26, 1.31)	0.42 (−0.32, 1.19)	0.49 (−0.26, 1.36)	0.52 (−0.07, 1.20)	0.39 (−0.13, 1.01)
*d*_*AC*_	0.84 (0.39, 1.34)	0.81 (0.41, 1.26)	0.83 (0.39, 1.34)	0.82 (0.42, 1.26)	0.84 (0.35, 1.40)	0.81 (0.38, 1.28)	0.80 (0.39, 1.24)
*d*_*AD*_	1.09 (0.26, 2.00)	1.05 (0.30, 1.87)	1.11 (0.3, 1.99)	1.02 (0.19, 1.92)	1.16 (0.19, 2.20)	1.12 (0.48, 1.80)	0.92 (0.29, 1.70)
*τ*	0.82 (0.55, 1.27)	0.73 (0.50, 1.09)	‐	‐	‐	‐	‐
*τ*_*AB*_	‐	‐	0.83 (0.55, 1.31)	0.70 (0.48, 1.04)	0.81 (0.20, 1.63)	0.72 (0.13, 1.24)	0.45 (0.15, 1.20)
*τ*_*AC*_	‐	‐	0.83 (0.55, 1.31)	0.70 (0.48, 1.04)	0.93 (0.60, 1.50)	0.79 (0.52, 1.24)	0.77 (0.51, 1.22)
*τ*_*AD*_	‐	‐	0.83 (0.55, 1.31)	0.70 (0.48, 1.04)	1.12 (0.48, 1.89)	0.77 (0.18, 1.30)	0.63 (0.17, 1.75)
*τ*_*BC*_	‐	‐	0.73 (0.41, 1.31)	0.97 (0.67, 1.45)	0.73 (0.15, 1.57)	0.28 (0.06, 0.97)	0.54 (0.16, 1.28)
*τ*_*BD*_	‐	‐	0.73 (0.41, 1.31)	0.97 (0.67, 1.45)	0.87 (0.17, 1.83)	0.25 (0.06, 0.91)	0.49 (0.15, 1.50)
*τ*_*CD*_	‐	‐	0.73 (0.41, 1.31)	0.97 (0.67, 1.45)	0.89 (0.18, 1.84)	0.28 (0.06, 1.06)	0.56 (0.18, 1.59)

a No intervention (A); self‐help (B); individual counselling (C); group counselling (D).

When assuming proportional heterogeneity variances (approach 2), the impact of using informative rather than vague priors is different because we now assume the heterogeneity variance for active vs active comparisons to be larger than that for active vs control comparisons, based on external evidence (Table S1). Pooling information across three comparisons leads to a narrower interval for the heterogeneity standard deviation than under than the unequal variances model (approach 3), and for the active vs active comparisons, the central estimate for heterogeneity is now much further from the prior median.

When using scaled inverse Wishart distributions (approach 4), the prior correlation between two different heterogeneity variances (on the log scale) is 0.71 when the two comparisons include a common treatment and 0.64 otherwise. These correlations are substantially higher than under approach 3 where the prior correlation is 0.39 when the two comparisons include a common treatment or 0 otherwise, and therefore, more information on heterogeneity is borrowed across comparisons and the heterogeneity variance results are closer. Changes to estimated heterogeneity variances have resulted in changes to the central estimates and intervals for the log odds ratios. In particular, the heterogeneity variance for comparison AB is much lower under approach 4, and this has led to much narrower intervals for the corresponding log odds ratio and a shift in the central estimates towards the null effect.

The conclusions about relative effectiveness of the four treatments are the same under all models fitted.

### 4.2 Network of treatments for localised prostate cancer

**Figure 2:**
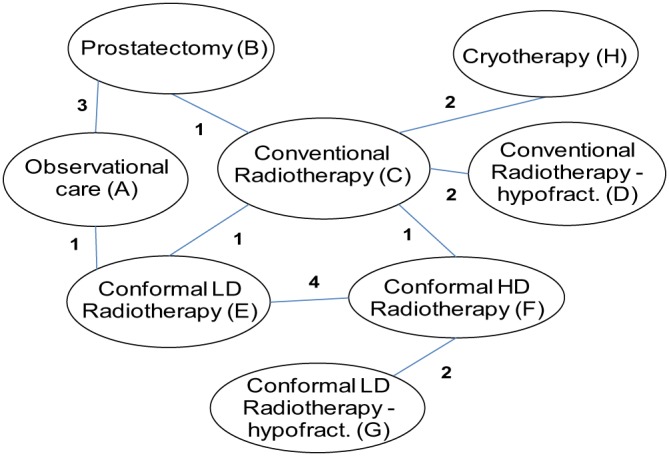
Network meta‐analysis comparing treatments for localised prostate cancer. LD indicates low dose and HD indicates high dose [Colour figure can be viewed at wileyonlinelibrary.com]

As a second illustrative example, we reanalysed a network meta‐analysis comparing eight treatments for localised prostate cancer, including five different RT regimes: observational management (A), prostatectomy (B), conventional RT (C), conventional RT hypofractionated (D), conformal low‐dose RT (E), conformal high‐dose RT (F), conformal low‐dose RT hypofractionated (G), and cryotherapy (H) (Figure 2).^20^ The outcome is all‐cause mortality and data from 17 trials are included (Table 5).

**Table 5:** Data from network meta‐analysis comparing eight treatments^a^ for localised prostate cancer with respect to all‐cause mortality^20^

Study	Arm 1	Events/Total in Arm 1	Arm 2	Events/Total in Arm 2
1	A	36/46	B	36/49
2	A	183/367	B	171/364
3	A	106/348	B	83/347
4	A	74/107	E	64/107
5	B	15/46	C	19/49
6	C	17/109	D	15/108
7	C	89/470	D	77/466
8	C	40/111	E	39/114
9	C	17/150	F	15/151
10	C	3/31	H	3/33
11	C	13/114	H	12/117
12	E	10/197	F	8/195
13	E	45/421	F	49/422
14	E	13/153	F	13/153
15	E	49/331	F	43/333
16	F	1/44	G	1/47
17	F	3/85	G	2/83

a Observational management (A); prostatectomy (B); conventional radiotherapy (C); conventional radiotherapy hypofractionated (D); conformal low‐dose radiotherapy (E); conformal high‐dose radiotherapy (F); conformal low‐dose radiotherapy hypofractionated (G); cryotherapy (H).

In the equal variances model (approach 1), the central estimate for heterogeneity is low at 0.09 (95% credible interval 0.005, 0.36). As an informative prior for *τ*^2^, we chose a log‐Normal(−4.28, 1.61^2^) prior for *τ*^2^, which represents a predictive distribution for heterogeneity in meta‐analyses with an all‐cause mortality outcome. When this prior was specified, the 95% credible interval for *τ*^2^ narrowed, which caused small changes to the central estimates for the log odds ratios and narrowing of their 95% credible intervals (Table 6).

**Table 6:** Comparison of eight treatments^a^ for localised prostate cancer when assuming equal, proportional, or unequal heterogeneity variances: posterior medians and 95% credible intervals for log odds ratios and between‐trial standard deviations,^b^ for the outcome of all‐cause mortality

	Approach 1:	Approach 1:	Approach 2:	Approach 2:	Approach 3:	Approach 3:	Approach 4:
	Equal Variances	Equal Variances	Proportional	Proportional	Unequal Variances	Unequal Variances	Scaled Inverse
	(Vague Prior)	(Inf. Prior)	Variances (Vague Priors)	Variances (Inf. Priors)	(Vague Priors)	(Inf. Priors)	Wishart (Inf. Prior)
*d*_*AB*_	−0.24 (−0.53, 0.03)	−0.23 (−0.48, 0.01)	−0.23 (−0.53, 0.05)	−0.23 (−0.48, 0.01)	−0.25 (−1.18, 0.61)	−0.23 (−0.53, 0.04)	−0.23 (−0.50, 0.03)
*d*_*AC*_	−0.17 (−0.80, 0.45)	−0.15 (−0.75, 0.43)	−0.16 (−0.77, 0.48)	−0.16 (−0.76, 0.42)	−0.15 (−1.47, 1.21)	−0.16 (−0.75, 0.47)	−0.17 (−0.75, 0.42)
*d*_*AD*_	−0.33 (−1.06, 0.40)	−0.30 (−1.01, 0.37)	−0.31 (−1.04, 0.43)	−0.32 (−1.04, 0.37)	−0.31 (−2.22, 1.63)	−0.31 (−1.03, 0.43)	−0.33 (−1.02, 0.36)
*d*_*AE*_	−0.32 (−0.86, 0.23)	−0.31 (−0.82, 0.22)	−0.3 (−0.86, 0.26)	−0.32 (−0.85, 0.18)	−0.32 (−1.51, 1.01)	−0.30 (−0.84, 0.25)	−0.32 (−0.83, 0.22)
*d*_*AF*_	−0.35 (−0.98, 0.24)	−0.35 (−0.91, 0.25)	−0.34 (−0.95, 0.28)	−0.36 (−0.95, 0.21)	−0.36 (−1.66, 1.04)	−0.34 (−0.95, 0.26)	−0.35 (−0.94, 0.24)
*d*_*AG*_	−0.68 (−2.45, 0.99)	−0.64 (−2.29, 0.91)	−0.69 (−2.54, 0.9)	−0.76 (−2.53, 1.03)	−0.68 (−3.29, 1.94)	−0.68 (−2.46, 1.03)	−0.67 (−2.47, 0.98)
*d*_*AH*_	−0.29 (−1.30, 0.76)	−0.27 (−1.23, 0.71)	−0.28 (−1.32, 0.75)	−0.27 (−1.25, 0.72)	−0.26 (−2.4, 1.89)	−0.26 (−1.26, 0.73)	−0.30 (−1.25, 0.69)
*τ*	0.09 (0.005, 0.36)	0.08 (0.02, 0.23)	‐	‐	‐	‐	‐
*τ*_*AB*_	‐	‐	0.10 (0.003, 0.39)	0.08 (0.03, 0.21)	0.51 (0.08, 1.67)	0.11 (0.03, 0.37)	0.08 (0.03, 0.28)
*τ*_*AC*_	‐	‐	0.10 (0.003, 0.39)	0.08 (0.03, 0.21)	0.62 (0.10, 1.71)	0.11 (0.03, 0.36)	0.08 (0.03, 0.31)
*τ*_*AD*_	‐	‐	0.10 (0.003, 0.39)	0.08 (0.03, 0.21)	0.81 (0.13, 1.85)	0.11 (0.03, 0.40)	0.08 (0.03, 0.32)
*τ*_*AE*_	‐	‐	0.10 (0.003, 0.39)	0.08 (0.03, 0.21)	0.55 (0.09, 1.67)	0.11 (0.03, 0.35)	0.08 (0.03, 0.30)
*τ*_*AF*_	‐	‐	0.10 (0.003, 0.39)	0.08 (0.03, 0.21)	0.57 (0.10, 1.68)	0.11 (0.03, 0.35)	0.08 (0.03, 0.30)
*τ*_*AG*_	‐	‐	0.10 (0.003, 0.39)	0.08 (0.03, 0.21)	0.93 (0.15, 1.89)	0.12 (0.03, 0.45)	0.08 (0.03, 0.32)
*τ*_*AH*_	‐	‐	0.10 (0.003, 0.39)	0.08 (0.03, 0.21)	0.87 (0.14, 1.87)	0.11 (0.03, 0.44)	0.08 (0.03, 0.32)

a Observational management (A); prostatectomy (B); conventional radiotherapy (C); conventional radiotherapy hypofractionated (D); conformal low‐dose radiotherapy (E); conformal high‐dose radiotherapy (F); conformal low‐dose radiotherapy hypofractionated (G); cryotherapy (H).

b For brevity, this Table reports a partial set of between‐trial standard deviations, whereas those for the remaining comparisons are reported in the Supplementary Material (Table S2).

Next, we declared separate priors for heterogeneity variances corresponding to active vs active comparisons and active vs control comparisons (approach 2). The data‐based predictive distributions are log‐Normal(−3.50, 1.26^2^) for nonpharmacological vs nonpharmacological comparisons and log‐Normal(−4.17, 1.55^2^) for nonpharmacological vs control comparisons (Table S1), which have medians of 0.17 and 0.12, respectively. Under this model, heterogeneity is estimated as slightly higher for the active vs active comparisons (Tables 6 and S2), influenced strongly by the prior distribution. The central estimates and 95% credible intervals for the log odds ratios are similar to those obtained under the equal variances model when using a single informative prior.

In this data set, many treatment comparisons are not directly informed by trial data, whereas those with data are informed by only 1 or 2 trials. Imprecision is therefore high for all heterogeneity variances in the unequal variances model (approach 3), when declaring vague Uniform(0, 2) priors for the *τ*_*k*_. The implied priors for the contrast‐specific standard deviations *τ*_*kl*_ have a higher median of 1.21 and 95% range of (0.25, 2.08) than under a Uniform(0, 2) prior, which also leads to higher central estimates and wider intervals for the *τ*_*kl*_ than in the equal variances model. Next, we specified log‐Normal(−4.83, 1.69^2^) priors for the $\tau_{k}^{2}$ (Table 2), to imply priors with mean and standard deviation matching those of the chosen log‐Normal(−4.28, 1.61^2^) priors for the $\tau_{\mathrm{kl}}^{2}$. The informative prior is very influential in the unequal variances model; posterior medians for all heterogeneity standard deviations are close to the prior median of 0.12, and their 95% credible intervals are similar to each other and to the prior 95% interval of (0.03, 0.57). The 95% credible intervals for the log odds ratios are substantially narrower when informative priors are used, whereas the central estimates are little changed.

As for the smoking cessation example, the prior correlation between two different heterogeneity variances (on the log scale) is 0.71 when there is a common comparator and 0.64 otherwise under approach 4, compared to 0.39 when there is a common comparator and 0 otherwise under approach 3. In the prostate cancer example, borrowing more information on heterogeneity across comparisons has resulted in narrower 95% credible intervals for the heterogeneity standard deviations and a small shift in the central estimates away from the prior median. These changes have led to slightly narrower 95% credible intervals for the log odds ratios.

In this network, there is no evidence of differences between the eight treatments; this conclusion is the same under all models fitted.

## 5 DISCUSSION

External evidence on the likely magnitude of heterogeneity variances has been published for various meta‐analysis settings, based on the Cochrane Database of Systematic Reviews. We have explored how to use this evidence to inform estimation of multiple heterogeneity variances in network meta‐analysis. If it is considered realistic to assume heterogeneity to be equal across all treatment comparisons, using an informative prior for the common heterogeneity variance in approach 1 is straightforward. Approach 2 allows us to specify separate informative priors for different intervention comparison types, under the assumption of equal heterogeneity within each comparison type and fixed heterogeneity ratios across types. To allow heterogeneity variances to be unequal across all treatment comparisons, we can use approaches 3 or 4. Approach 3 assumes minimal correlation between different heterogeneity variances, and thus, very little information on heterogeneity is borrowed across comparisons. Under this approach, inference about heterogeneity for comparisons informed by few studies is based primarily on the prior distribution. When using approach 4, higher prior correlations are assumed between the multiple heterogeneity variances, and thus, information is borrowed across comparisons. This approach provides a compromise between assuming all heterogeneity variances to be equal in approach 1 and assuming them to be unequal and minimally correlated in approach 3. The amount of information borrowed across comparisons in approach 4 could be increased by using an inverse Wishart distribution with higher degrees of freedom. Increasing the amount of borrowing could be particularly useful in a sparse network in which few trials inform each comparison, such as the prostate cancer treatments example.

Throughout this paper, we presented models assuming consistency across the network meta‐analysis, meaning that indirect evidence on treatment differences is assumed to agree with direct evidence. However, the informative priors proposed for heterogeneity could be used in inconsistency models, which relax this assumption. If a consistency model is used in a network in which inconsistency is present, the heterogeneity variances model both between‐trial heterogeneity and inconsistency, and the data‐based informative priors may not then be appropriate. The same approaches to specifying informative priors for heterogeneity could also be used in network meta‐regression models. However, it is unlikely that any prior evidence would be available for the residual heterogeneity remaining after adjustment for a specific combination of study covariates. If empirical distributions based on random‐effects meta‐analyses are used to inform a prior, this could be viewed as a conservative choice, which supports larger values of heterogeneity than necessary. We have presented models for binary outcomes, but the approaches can be applied directly to network meta‐analyses evaluating continuous outcomes on the standardised mean difference scale, for which relevant data‐based priors for heterogeneity are available.^6^

The approaches proposed have some limitations. When using approach 2, we should check that the separate priors chosen result in a positive semidefinite covariance matrix for the vector of contrasts with the reference treatment. In practice, however, we expect that using priors that do not guarantee positive semidefiniteness will be problematic only in networks that include a trial comparing five treatments or more because the covariance matrix assumed may be invalid for that trial. If a network comparing five treatments or more includes only pairwise, three‐arm and four‐arm trials, the covariance matrix for any one trial will be positive semidefinite, although the covariance matrix across the whole network may not be. Approaches 3 and 4 are based on approximating proposed forms of prior to target priors, using the method of moments, so these approaches involve using priors that are not identical to the published data‐based priors. Amongst all the approaches considered, implementation is particularly complicated for approach 3, so this approach may be less useful than the others in practice.

Incorporating external evidence about expected between‐study heterogeneity will not be appropriate in all network meta‐analyses. In some networks, an assumption of common heterogeneity across comparisons may be entirely plausible and the combined set of studies may then provide sufficient precision for estimating heterogeneity. In other networks, even where heterogeneity is expected to differ somewhat amongst comparisons, it could be preferable to make an assumption of common heterogeneity and borrow information internally across the network rather than to borrow information from external sources. The choice between different possible approaches should be informed by the similarity of the treatment comparisons in the network and the relevance of available external data. We note that network structure does not affect the potential for updating prior distributions for heterogeneity with evidence and, therefore, should not influence choice between approaches. Indirect evidence arising within a loop of treatments (for example, ABC in Figure 1) informs estimation of average treatment differences for each comparison in the loop but does not inform the estimation of heterogeneity variances because the heterogeneity variance matrix includes a separate parameter for every comparison in the network.

We have not included an approach assuming exchangeability for the heterogeneity variances across treatment comparisons. This could be useful in allowing more information to be borrowed amongst comparisons, but it is not clear how to assume exchangeability while meeting the requirement of second‐order consistency. It would also be desirable to find an approach allowing unequal heterogeneity variances across treatment comparisons, for which different informative priors may be chosen. In approach 4, it is not possible to match moments to target priors separately for different heterogeneity variances in the network. In approach 3, matching moments to multiple data‐based priors would be possible in principle and would require us to solve an extended set of nonlinear equations. However, as we were able to solve Equation (4) once and then present informative priors facilitating implementation of approach 3 in any future network, a different set of extended equations would need to be solved separately for each individual network, which would be very unappealing in practice.

Thorlund et al^21^ explored modelling approaches for heterogeneity variances in network meta‐analysis. Data‐based informative priors were used in a model allowing unrestricted unequal heterogeneity variances across comparisons, in which second‐order consistency was not guaranteed. Separately, they implemented a second‐order consistency model based on the Lu and Ades approach and a model assuming exchangeability of heterogeneity variances, using vague priors for heterogeneity in both. Thorlund et al^21^ recommended improving estimation of heterogeneity variances in network meta‐analysis by incorporating external information through data‐based priors or by borrowing information across the network through assuming exchangeability or second‐order consistency. Our work goes further by proposing approaches for using data‐based priors for heterogeneity while also ensuring that second‐order consistency holds. Ren et al^22^ proposed truncating empirical data‐based log‐normal distributions for heterogeneity using elicited opinion and reducing prior support for extremely high values. Truncated log‐normal priors could be used directly in approach 1, and could also be used in approach 2 if priors for different intervention comparison types were truncated at a common quantile. Approaches 3 and 4 assume log‐normality and would need to be revised if truncated distributions were used.

In conclusion, incorporating informative priors in network meta‐analysis models assuming equal heterogeneity variances is straightforward, but this assumption is not always plausible. We have proposed several approaches for using data‐based priors for multiple unequal heterogeneity variances. If it is reasonable to assume equality within intervention comparison types and fixed heterogeneity ratios across types, we recommend approach 2. If it is desired to model all heterogeneity variances in the network as unequal, we recommend approach 4, which is simpler to implement than approach 3. These methods would be useful in sparse data sets and may increase precision for estimating treatment differences.

## Supporting information

SIM_8044‐Supp‐0001‐Informative_priors_for_network_MA_revised_supplementary.docx
