# Supplementary material for: Incorporating external evidence on between‐trial heterogeneity in network meta‐analysis
Source: Stat Med. 2018 Nov 28;38(8):1321–35. doi: 10.1002/sim.8044 (PMC6492109; doi:10.1002/sim.8044)
Supplement: Supplementary file 1 — SIM_8044‐Supp‐0001‐Informative_priors_for_network_MA_revised_supplementary.docx [file SIM-38-1321-s001.docx]

# Supplementary material

## A1 Exploring positive semi-definiteness under approach 2

Here, we explore the conditions under which separate priors for $\tau_{kl}^{2}$ of the form (2) result in positive semi-definiteness for $\Sigma$.

In a network including three treatments, the vector of contrasts with the reference treatment 0 in study *j* is $\mathbf{\delta}_{j}=\left(\delta_{j01},\delta_{j02}\right)\sim N\left(\mathbf{d},\mathbf{\Sigma}_{2}\right)$ and

$$\Sigma_{2}=\tau^{2}\left(\begin{matrix} \exp\left(m_{01}\right) & \frac{1}{2}\left\{\exp\left(m_{01}\right)+\exp\left(m_{02}\right)-\exp\left(m_{12}\right)\right\} \\ \frac{1}{2}\left\{\exp\left(m_{01}\right)+\exp\left(m_{02}\right)-\exp\left(m_{12}\right)\right\} & \exp\left(m_{02}\right) \end{matrix}\right)$$

$\mathbf{\Sigma}_{2}$ is positive semi-definite if and only if

$\frac{1}{4}\left\{\exp\left(m_{01}\right)+\exp\left(m_{02}\right)-\exp\left(m_{12}\right)\right\}^{2}\leq\exp\left(m_{01}\right)\exp\left(m_{02}\right)$ (S1)

We find by checking numerically that condition (2) holds for any combination of the priors presented in Table S1.

We now consider the general case of a network including *p*+1 treatments. Without loss of generality, we assume the following:

- The reference treatment is, in order of preference: the placebo or control treatment (if there is one), a pharmacological treatment (if there are any), or a non-pharmacological treatment.
- The first *q* of the *p* active treatments are pharmacological.
- The remaining *p-q* active treatments are non-pharmacological.

Then, we can write $\mathbf{\Sigma}_{p}$ as a partitioned matrix:

$\mathbf{\Sigma}_{p}=\tau^{2}\left(\begin{matrix} A_{q\times q} & B_{q\times(p-q)} \\ B_{q\times(p-q)}^{T} & C_{(p-q)\times(p-q)} \end{matrix}\right)$ (S2)

where:

$$A_{q\times q}=\left\{\exp\left(m_{11}\right)/2\right\}\mathbf{I}_{l}+\left\{\exp\left(m_{01}\right)-\exp\left(m_{11}\right)/2\right\}\mathbf{1}_{q}\mathbf{1}_{q}^{T}$$

$B_{q\times\left(p-q\right)}=\frac{1}{2}\left\{\exp\left(m_{01}\right)+\exp\left(m_{02}\right)-\exp\left(m_{12}\right)\right\}\mathbf{1}_{q}\mathbf{1}_{p-q}^{T}$ (S3)

$$C_{\left(p-q\right)\times\left(p-q\right)}=\left\{\exp\left(m_{22}\right)/2\right\}\mathbf{I}_{p-q}+\left\{\exp\left(m_{02}\right)-\exp\left(m_{22}\right)/2\right\}\mathbf{1}_{p-q}\mathbf{1}_{p-q}^{T}$$

In the above matrices (S3), the prior means for different types of contrast are referred to as $m_{01}$ for pharmacological vs. reference (which may be placebo/control, pharmacological or non-pharmacological), $m_{02}$ for non-pharmacological vs. reference, $m_{11}$ for pharmacological vs. pharmacological, $m_{22}$ for non-pharmacological vs. non-pharmacological, and $m_{12}$ for pharmacological vs. non-pharmacological.

Using the partitioned matrix (S2), we have checked whether $\mathbf{\Sigma}_{p}$ is positive semi-definite for networks including different numbers of pharmacological and non-pharmacological treatments, for up to *p*=100 active treatments, for the priors presented in Table S1. The results are summarised at the end of section 3.2.

## A2 Derivation of matching moments equations (4) in approach 3

To find a suitable log-Normal prior for the $\tau_{k}^{2}$, we match the moments of the implied prior for $\tau_{kl}^{2}$ based on expression (3) with the known moments of a chosen data-based informative prior $\tau_{kl}^{2}\sim\text{log-N}\left(m_{D},s_{D}^{2}\right)$. The data-based informative prior has mean $e^{m_{D}}e^{s_{D}^{2}/2}$ and variance $e^{2m_{D}}e^{s_{D}^{2}}\left(e^{s_{D}^{2}}-1\right)$.

We will now derive the moments of the implied prior for $\tau_{kl}^{2}$ under expression (3). We assume $\text{log-Normal}\left(m,s^{2}\right)$ priors for $\tau_{k}^{2}$ and $\tau_{l}^{2}$. Independently of $\tau_{k}^{2}$ and $\tau_{l}^{2}$, we assume a generic prior distribution for the correlation $\rho_{kl}$ in (3), with mean $m_{\rho}$ and variance $s_{\rho}^{2}$. Under these assumptions, we would like to find the implied mean and variance for $\tau_{kl}^{2}=\tau_{k}^{2}+\tau_{l}^{2}-2\rho_{kl}\tau_{k}\tau_{l}$.

First, we find the expectation of $\tau_{kl}^{2}$. We will make use of a general result^1^ that if $X\sim\log\text{-N}\left(\mu,\sigma^{2}\right)$, then $X^{a}\sim\log\text{-N}\left(a\mu,a^{2}\sigma^{2}\right)$. We apply this result to find that $\tau_{k},\tau_{l}\sim\log\text{-N}\left(m/2,s^{2}/4\right)$, since $\tau_{k}^{2},\tau_{l}^{2}\sim\log-N\left(m,s^{2}\right)$ , and therefore $E\left(\tau_{k}\right)=E\left(\tau_{l}\right)=e^{m/2}e^{s^{2}/8}$, because the mean of a $\log\text{-N}\left(\mu,\sigma^{2}\right)$ distribution is $e^{\mu}e^{\sigma^{2}/2}$.^1^ Assuming independence for $\rho_{kl}$, $\tau_{k}$ and $\tau_{l}$, we then calculate the following:

$$\begin{matrix} E\left(\tau_{kl}^{2}\right)=\text{E}\left(\tau_{k}^{2}\right)+\text{E}\left(\tau_{l}^{2}\right)-2\text{E}\left(\rho_{kl}\right)\text{E}\left(\tau_{k}\right)\text{E}\left(\tau_{l}\right) \\ =e^{m}e^{s^{2}/2}+e^{m}e^{s^{2}/2}-2m_{\rho}\left(e^{m/2}e^{\sigma^{2}/8}\right)^{2}=2e^{m}e^{s^{2}/4}\left(e^{s^{2}/4}-m_{\rho}\right) \end{matrix}$$

Next, we find the variance of $\tau_{kl}^{2}$. We first use our knowledge that $\tau_{k},\tau_{l}\sim\log\text{-N}\left(m/2,s^{2}/4\right)$, and find by summing independent normal distributions for $\log\left(\tau_{k}\right)$ and $\log\left(\tau_{l}\right)$ that $\tau_{k}\tau_{l}\sim\log\text{-N}\left(m,s^{2}/2\right)$. For convenience, we will write $Y_{kl}=\tau_{k}\tau_{l}$ . The variance of $\tau_{kl}^{2}$ is calculated as follows:

$$\begin{matrix} Var\left(\tau_{kl}^{2}\right)=Var\left(\tau_{k}^{2}+\tau_{l}^{2}-2\rho_{kl}\tau_{k}\tau_{l}\right) \\ =Var\left(\tau_{k}^{2}+\tau_{l}^{2}\right)+4Var\left(\rho_{kl}Y_{kl}\right)-4Cov\left(\tau_{k}^{2}+\tau_{l}^{2},\rho_{kl}Y_{kl}\right) \\ =Var\left(\tau_{k}^{2}+\tau_{l}^{2}\right)+4Var\left(\rho_{kl}Y\right)-4Cov\left(\tau_{k}^{2},\rho_{kl}Y_{kl}\right)-4Cov\left(\tau_{l}^{2},\rho_{kl}Y_{kl}\right) \end{matrix}$$

The variance of $\tau_{k}^{2}+\tau_{l}^{2}$ is $2e^{2m}e^{s^{2}}\left(e^{s^{2}}-1\right)$ , using the standard formula for the variance of a log-normal distribution. To calculate the variance of $\rho_{kl}Y_{kl}$, we use the formula^2^ for the variance of a product of two independent random variables and obtain the following:

$$\begin{matrix} Var\left(\rho_{kl}Y_{kl}\right)=E\left(\rho_{kl}\right)^{2}Var\left(Y_{kl}\right)+E\left(Y_{kl}\right)^{2}Var\left(\rho_{kl}\right)+Var\left(\rho_{kl}\right)Var\left(Y_{kl}\right) \\ =m_{\rho}^{2}e^{2m}e^{s^{2}/2}\left(e^{s^{2}/2}-1\right)+\left(e^{m}e^{s^{2}/4}\right)^{2}s_{\rho}^{2}+s_{\rho}^{2}e^{2m}e^{s^{2}/2}\left(e^{s^{2}/2}-1\right) \end{matrix}$$

Again applying the general result that $X^{a}\sim\log\text{-N}\left(a\mu,a^{2}\sigma^{2}\right)$ if $X\sim\log\text{-N}\left(\mu,\sigma^{2}\right)$, we calculate $Cov\left(\tau_{k}^{2},\rho_{kl}Y_{kl}\right)$ below, assuming independence for $\rho_{kl}$, $\tau_{k}$ and $\tau_{l}$, and for $\rho_{kl}$ and $Y_{kl}$, and using the definition $Cov\left(X_{1},X_{2}\right)=E\left(X_{1}X_{2}\right)-E\left(X_{1}\right)E\left(X_{2}\right)$:

$$\begin{matrix} Cov\left(\tau_{k}^{2},\rho_{kl}Y_{kl}\right)=E\left(\rho_{kl}\right)E\left(\tau_{k}^{3}\right)E\left(\tau_{l}\right)-E\left(\tau_{k}^{2}\right)E\left(\rho_{kl}\right)E\left(Y_{kl}\right) \\ =m_{\rho}\left(e^{3m/2}e^{9s^{2}/8}\right)\left(e^{m/2}e^{s^{2}/8}\right)-m_{\rho}\left(e^{m}e^{s^{2}/2}\right)\left(e^{m}e^{s^{2}/4}\right) \end{matrix}$$

Then, by algebraic manipulation, we find the following expression for the variance of $\tau_{kl}^{2}$:

$$\text{Var}\left(\tau_{kl}^{2}\right)=2e^{2m}e^{s^{2}}\left(e^{s^{2}}-1+2s_{\rho}^{2}\right)+4m_{\rho}^{2}e^{2m}e^{s^{2}/2}\left(e^{s^{2}/2}-1\right)-8m_{\rho}e^{2m}e^{3s^{2}/4}\left(e^{s^{2}/2}+1\right)$$

We now equate the derived mean and variance of the implied prior for $\tau_{kl}^{2}=\tau_{k}^{2}+\tau_{l}^{2}-2\rho_{kl}\tau_{k}\tau_{l}$ to the mean and variance of the data-based informative prior, and obtain expression (4).

## A3 Priors chosen for the correlations in approach 3

Lu and Ades^3^ suggested using Uniform priors for the or Beta priors for the in (5). However, specifying identical priors for the spherical parameters will lead to non-identical priors for the correlations $\rho_{kl}$, and hence also for the $\tau_{kl}^{2}$ through (3). To address this, we propose a modification to the model, based on the assumption that, a priori, the relative order of the treatments (excluding the reference treatment) is unimportant and that the correlations are exchangeable. To implement this, we propose to declare auxiliary priors for a set of correlations using a spherical parameterisation (as shown in (5) for three treatments), and then permute the order of the treatments. For example, in a network with three treatments, we declare priors for the $\rho_{kl}$ as follows:

${\rho^{'}}_{12}=\text{cos}\left( \phi_{12} \right)$ (S4)

$${\rho^{'}}_{13}=\text{cos}\left( \phi_{13} \right)$$

$${\rho'}_{23}=\text{cos}\left( \phi_{12} \right)\text{cos}\left( \phi_{13} \right)+\text{sin}\left( \phi_{12} \right)\text{sin}\left( \phi_{13} \right)\text{cos}\left( \phi_{23} \right)$$

$$\rho_{12}= {\rho^{'}}_{\pi(1)\pi(2)}$$

$$\rho_{13}= {\rho^{'}}_{\pi\left( 1 \right)\pi\left( 3 \right)}$$

$$\rho_{23}= {\rho^{'}}_{\pi(2)\pi(3)}$$

where *π* is a permutation of {1, 2, 3}. By setting a flat prior for *π* over all the possible permutations (six in this case), we ensure that all three correlations $\rho_{kl}$ have identical prior distributions and therefore that the heterogeneity variances $\tau_{kl}^{2}$ also have identical prior distributions, if identical priors are declared for the $\tau_{k}^{2}$ in (3). A drawback to this approach is that the mean $\mu_{\rho}$ and variance $\sigma_{\rho}^{2}$ of the prior distribution for $\rho_{kl}$, needed to match moments in (4), are harder to calculate. These moments can be obtained via numerical integration or Monte Carlo simulation and will differ for different network sizes.

## A4 Choice of inverse Wishart distribution in approach 4

In approach 4, our choice for the value $S_{kl}$in the matrix $\mathbf{S}$ is determined by considering how to ensure symmetry across treatment contrasts.

Under a standard inverse Wishart distribution, $\mathbf{\Sigma}^{-1}\sim Wishart\left(\mathbf{S},t\right)$, heterogeneity variances representing contrasts with the reference treatment are the diagonal elements, $\Sigma_{kk}$, which follow an inverse gamma distribution: $\Sigma_{kk}\sim IG\left(\left(t-p+1\right)/2,S_{kk}/2\right)$ ^4^. We will work out the distribution implied for the heterogeneity variances representing contrasts between two non-reference treatments *k* and *l*, given by $\Sigma_{kk}+\Sigma_{ll}-2\Sigma_{kl}$. Let $\mathbf{e}_{k}$ be a row vector of dimension *p* with a 1 in position *k* and zeroes elsewhere. We can write $\Sigma_{kk}+\Sigma_{ll}-2\Sigma_{kl}$ as $\left(\mathbf{e}_{k}-\mathbf{e}_{l}\right)\mathbf{\Sigma}\left(\mathbf{e}_{k}-\mathbf{e}_{l}\right)^{T}$. We make use of a known result for the distribution of an inverse Wishart distribution transformed to a scalar ^5^: $\left(\mathbf{v\Sigma v}^{T}\right)^{-1}\sim IG\left(\left(t-p+1\right)/2,\mathbf{vSv}^{T}/2\right)$, where $\mathbf{\Sigma}^{-1}\sim Wishart\left(\mathbf{S},t\right)$, $\mathbf{\Sigma}$ is of dimension *pxp*, and $\mathbf{v}$ is a row vector of length *p*. Using this result, we find that $\Sigma_{kk}+\Sigma_{ll}-2\Sigma_{kl}$ is distributed as $IG\left(\left(t-p+1\right)/2,\left(S_{kk}+S_{ll}-2S_{kl}\right)/2\right)$. By comparing the implied distributions for the $\Sigma_{kk}$ and the $\Sigma_{kk}+\Sigma_{ll}-2\Sigma_{kl}$, we find that these will be identical across treatments *k* and *l* if all $S_{kk}$ are equal, and if all $S_{kl}$ are equal to $S_{kk}/2$. When using a scaled inverse Wishart distribution $\mathbf{\Sigma}^{-1}\sim\mathbf{M}/\lambda$, where $\mathbf{M}\sim Wishart\left(\mathbf{S},t\right)$ and $\log\left(\lambda\right)\sim Normal\left(m_{\lambda},s_{\lambda}^{2}\right)$, this condition is unchanged.

## A5 WinBUGS code for implementing approaches 1 to 4

## Approach 1

The WinBUGS code below implements approach 1 for the smoking cessation network, with a log-Normal(-2.75,1.61^2^) prior for the heterogeneity variance $\tau^{2}$. This code has been adapted from code published by Dias et al.^6^.

model{

for(i in 1:NS) {

w[i,1] <- 0

delta[i,t[i,1]] <- 0

mu[i] ~ dnorm(0,.0001) # Fixed study effects

for (k in 1:na[i]) {

r[i,k] ~ dbin(p[i,t[i,k]],n[i,k]) # Binomial likelihood for data

logit(p[i,t[i,k]])<-mu[i] + delta[i,t[i,k]] } # Model for log odds parameters

for (k in 2:na[i]) {

delta[i,t[i,k]] ~ dnorm(md[i,t[i,k]],precd[i,t[i,k]]) # Random treatment effects

md[i,t[i,k]] <- d[t[i,k]] - d[t[i,1]] + sw[i,k] # Mean of LOR distributions

precd[i,t[i,k]] <- prec *2*(k-1)/k # Precision of LOR distributions

w[i,k] <- (delta[i,t[i,k]] - d[t[i,k]] + d[t[i,1]]) # Adjustment, multi-arm RCTs

sw[i,k] <-sum(w[i,1:k-1])/(k-1) } # Cumulative adjustment, multi-arm RCTs

}

d[1]<-0

for (k in 2:NT) {d[k] ~ dnorm(0,.0001) } # Vague priors for basic parameters

tausq ~ dlnorm(-2.75,0.386) # Informative prior for heterogeneity

prec <- 1/tausq

tau <- sqrt(tausq)

# Defining pairwise ORs

for (c in 1:(NT-1))

{ for (k in (c+1):NT)

{ lor[c,k] <- d[k] - d[c]

log(or[c,k]) <- lor[c,k] } }

}

The smoking cessation data set is provided below, where r[,k], n[,k] and t[,k] represent the numbers of events, total numbers of patients and treatment received in arm k of each study, and na[] represents the number of arms in the study. NS and NT represent the total number of studies and treatments respectively.

r[,1] n[,1] r[,2] n[,2] r[,3] n[,3] t[,1] t[,2] t[,3] na[]

9 140 23 140 10 138 1 3 4 3

11 78 12 85 29 170 2 3 4 3

75 731 363 714 NA 1 1 3 NA 2

2 106 9 205 NA 1 1 3 NA 2

58 549 237 1561 NA 1 1 3 NA 2

0 33 9 48 NA 1 1 3 NA 2

3 100 31 98 NA 1 1 3 NA 2

1 31 26 95 NA 1 1 3 NA 2

6 39 17 77 NA 1 1 3 NA 2

79 702 77 694 NA 1 1 2 NA 2

18 671 21 535 NA 1 1 2 NA 2

64 642 107 761 NA 1 1 3 NA 2

5 62 8 90 NA 1 1 3 NA 2

20 234 34 237 NA 1 1 3 NA 2

0 20 9 20 NA 1 1 4 NA 2

8 116 19 149 NA 1 1 2 NA 2

95 1107 143 1031 NA 1 1 3 NA 2

15 187 36 504 NA 1 1 3 NA 2

78 584 73 675 NA 1 1 3 NA 2

69 1177 54 888 NA 1 1 3 NA 2

20 49 16 43 NA 1 2 3 NA 2

7 66 32 127 NA 1 2 4 NA 2

12 76 20 74 NA 1 3 4 NA 2

9 55 3 26 NA 1 3 4 NA 2

Data structure: list(NS=24,NT=4)

## Approach 2

The WinBUGS code below implements approach 2 for the smoking cessation network, with a log-Normal(-2.26,1.45^2^) prior for heterogeneity variances corresponding to non-pharmacological vs. non-pharmacological comparisons and a log-Normal prior for heterogeneity variances corresponding to non-pharmacological vs. control comparisons. This code has been adapted from code published by Dias et al.^6^.

model{

for(i in 1:NS) {

w[i,1] <- 0

delta[i,t[i,1]] <- 0

mu[i] ~ dnorm(0,.0001) # Fixed study effects

for (k in 1:na[i]) {

r[i,k] ~ dbin(p[i,t[i,k]],n[i,k]) # Binomial likelihood for data

logit(p[i,t[i,k]])<-mu[i] + delta[i,t[i,k]] } # Model for log odds parameters

for (k in 2:na[i]) {

delta[i,t[i,k]] ~ dnorm(md[i,t[i,k]],precd[i,t[i,k]]) # Random treatment effects

md[i,t[i,k]] <- d[t[i,k]] - d[t[i,1]] + sw[i,k] # Mean of LOR distributions

precd[i,t[i,k]] <- prec[contrast[i]]*2*(k-1)/k # Precision of LOR distributions

w[i,k] <- (delta[i,t[i,k]] - d[t[i,k]] + d[t[i,1]]) # Adjustment, multi-arm RCTs

sw[i,k] <-sum(w[i,1:k-1])/(k-1) } # Cumulative adjustment, multi-arm RCTs

}

d[1]<-0

for (k in 2:NT) {d[k] ~ dnorm(0,.0001) } # Vague priors for basic parameters

# Informative priors for heterogeneity

for(l in 1:2){ prec[l] <- 1/tausq[l] }

tausq[1] <- tausq.base*exp(-2.92) # Non-pharma vs. control contrasts

tau[1] <- sqrt(tausq[1])

tausq[2] <- tausq.base*exp(-2.26) # Non-pharma vs. non-pharma contrasts

tau[2] <- sqrt(tausq[2])

tausq.base ~ dlnorm(0,0.401) # Base distribution

# Note we have assumed equal variances for the priors, based on weighting the two different standard deviations by number of contrasts.

# Defining pairwise ORs

for (c in 1:(NT-1))

{ for (k in (c+1):NT)

{ lor[c,k] <- d[k] - d[c]

log(or[c,k]) <- lor[c,k] } }

}

The smoking cessation data set is as previously, with the addition of a variable contrast[], indicating whether contrasts were non-pharmacological vs. non-pharmacological or non-pharmacological vs. control:

r[,1] n[,1] r[,2] n[,2] r[,3] n[,3] t[,1] t[,2] t[,3] na[] contrast[]

9 140 23 140 10 138 1 3 4 3 1

11 78 12 85 29 170 2 3 4 3 2

75 731 363 714 NA 1 1 3 NA 2 1

2 106 9 205 NA 1 1 3 NA 2 1

58 549 237 1561 NA 1 1 3 NA 2 1

0 33 9 48 NA 1 1 3 NA 2 1

3 100 31 98 NA 1 1 3 NA 2 1

1 31 26 95 NA 1 1 3 NA 2 1

6 39 17 77 NA 1 1 3 NA 2 1

79 702 77 694 NA 1 1 2 NA 2 1

18 671 21 535 NA 1 1 2 NA 2 1

64 642 107 761 NA 1 1 3 NA 2 1

5 62 8 90 NA 1 1 3 NA 2 1

20 234 34 237 NA 1 1 3 NA 2 1

0 20 9 20 NA 1 1 4 NA 2 1

8 116 19 149 NA 1 1 2 NA 2 1

95 1107 143 1031 NA 1 1 3 NA 2 1

15 187 36 504 NA 1 1 3 NA 2 1

78 584 73 675 NA 1 1 3 NA 2 1

69 1177 54 888 NA 1 1 3 NA 2 1

20 49 16 43 NA 1 2 3 NA 2 2

7 66 32 127 NA 1 2 4 NA 2 2

12 76 20 74 NA 1 3 4 NA 2 2

9 55 3 26 NA 1 3 4 NA 2 2

Data structure: list(NS=24,NT=4)

## Approach 3

The OpenBUGS code below implements approach 3 for the smoking cessation network, with priors chosen to approximate log-Normal(-2.75,1.61^2^) priors for the $\tau_{kl}^{2}$. This code has been adapted from the code supplied by Lu and Ades ^3^.

model{

for(i in 1:NS) {

w[i,1] <- 0

delta[i,t[i,1]] <- 0

mu[i] ~ dnorm(0,.0001) # Fixed study effects

for (k in 1:na[i]) {

r[i,k] ~ dbin(p[i,t[i,k]],n[i,k]) # Binomial likelihood for data

logit(p[i,t[i,k]])<-mu[i] + delta[i,t[i,k]] } # Model for log odds parameters

for (k in 2:na[i]) {

delta[i,t[i,k]] ~ dnorm(md[i,t[i,k]],precd[i,t[i,k]]) # Random treatment effects

md[i,t[i,k]] <- d[t[i,k]] - d[t[i,1]] + sw[i,k] # Mean of LOR distributions

precd[i,t[i,k]] <- prec[t[i,1],t[i,k]]*2*(k-1)/k # Precision of LOR distributions

w[i,k] <- (delta[i,t[i,k]] - d[t[i,k]] + d[t[i,1]]) # Adjustment, multi-arm RCTs

sw[i,k] <-sum(w[i,1:k-1])/(k-1) } # Cumulative adjustment, multi-arm RCTs

}

d[1]<-0

for (k in 2:NT) {d[k] ~ dnorm(0,.0001) } # Vague priors for basic parameters

for(j in 1:3){

prec[j,j]<-1

for(k in (j+1):4){

prec[j,k]<-1/tausq[j,k]

prec[k,j]<-prec[j,k] }}

for(k in 1:4){

v.a[k]~dlnorm(-3.31,0.346) # Informative prior chosen to imply (approximately) chosen data-based prior for contrast heterogeneity variance

sd.a[k]<-sqrt(v.a[k])

}

pi.half<-1.5708

for(i in 1:3) {for(j in (i+1):4){

g[j,i]<-0

tausq[i,j]<-v.a[i]+v.a[j]-2*rho.star[i,j]*sd.a[i]*sd.a[j]

tau[i,j] <- sqrt(tausq[i,j]) }}

# Implementing random permutation

for(i in 1:4) { for(j in 1:4) {

rho[i,j]<-inprod(g[, i],g[, j])

rho.star[i,j]<- rho[ myorder[i], myorder[j] ] }}

for(i in 1:4) {

aux.u[i] ~ dunif(0, 1)

myorder[i] <- rank(aux.u[],i)

}

# Constructing entries of upper-triangular matrix for Cholesky decomposition

g[1,1]<-1

g[2,2]<-sin.a[1,2]

g[3,3]<-sin.a[1,3]*sin.a[2,3]

g[4,4]<-sin.a[1,4]*sin.a[2,4]*sin.a[3,4]

g[1,2]<-cos.a[1,2]

g[1,3]<-cos.a[1,3]

g[1,4]<-cos.a[1,4]

g[2,3]<-sin.a[1,3]*cos.a[2,3]

g[2,4]<-sin.a[1,4]*cos.a[2,4]

g[3,4]<-sin.a[1,4]*sin.a[2,4]*cos.a[3,4]

# Beta prior for cos(a[i,j])

for (i in 1:3) {

for (j in (i+1):4) {

cos.a[i,j] ~ dbeta(0.93,1.07) # From Table 3

sin2.a[i,j] <- 1 - pow(cos.a[i,j] , 2)

sin.a[i,j] <- pow(sin2.a[i,j] , 1/2) } }

# Defining pairwise ORs

for (c in 1:(NT-1))

{ for (k in (c+1):NT)

{ lor[c,k] <- d[k] - d[c]

log(or[c,k]) <- lor[c,k] } }

}

The data set is as presented for approach 1.

## Approach 4

The WinBUGS code below implements approach 4 for the smoking cessation network, with the prior for the scaling parameter $\lambda$ chosen to imply log-Normal(-2.75,1.61^2^) priors for the $\tau_{kl}^{2}$.

model{

for(i in 1:N) { logit(p[i]) <- mu[s[i]]+ delta[s[i],treat[i]] # Model for log odds parameters

r[i] ~ dbin(p[i],n[i]) # Binomial likelihood for data

}

for(j in 1:NS) { mu[j] ~ dnorm(0,.0001) # Fixed study effects

delta[j,1] <- 0

delta[j,2:NT] ~ dmnorm(d[2:NT], precd[1:NT-1,1:NT-1]) }

d[1]<-0

for (k in 2:NT) {d[k] ~ dnorm(0,.0001) } # Vague priors for basic parameters

covd[1:NT-1,1:NT-1] <- inverse(precd[1:NT-1,1:NT-1])

M[1:NT-1,1:NT-1] ~ dwish(R[1:NT-1,1:NT-1],4) # Wishart distribution with 4 df

lambda ~ dlnorm(-2.634,1.056) # Scaling parameter

tau[1] <- sqrt(covd[1,1])

tau[2] <- sqrt(covd[2,2])

tau[3] <- sqrt(covd[3,3])

tau[4] <- sqrt(covd[1,1]-2*covd[1,2]+covd[2,2])

tau[5] <- sqrt(covd[1,1]-2*covd[1,3]+covd[3,3])

tau[6] <- sqrt(covd[2,2]-2*covd[2,3]+covd[3,3])

for(l in 1:NT-1) {

for(m in 1:NT-1) {

precd[l,m] <- M[l,m]/lambda # Scaling applied to Wishart distribution

}

}

# Defining pairwise ORs

for (c in 1:(NT-1))

{ for (k in (c+1):NT)

{ lor[c,k] <- d[k] - d[c]

log(or[c,k]) <- lor[c,k] } }

}

A long form of the smoking cessation data set was used for this model:

s[] treat[] r[] n[]

1 1 79 702

1 2 77 694

2 1 18 671

2 2 21 535

3 1 8 116

3 2 19 149

4 1 75 731

4 3 363 714

5 1 2 106

5 3 9 205

6 1 58 549

6 3 237 1561

7 1 0 33

7 3 9 48

8 1 3 100

8 3 31 98

9 1 1 31

9 3 26 95

10 1 6 39

10 3 17 77

11 1 64 642

11 3 107 761

12 1 5 62

12 3 8 90

13 1 20 234

13 3 34 237

14 1 95 1107

14 3 143 1031

15 1 15 187

15 3 36 504

16 1 78 584

16 3 73 675

17 1 69 1177

17 3 54 888

18 1 9 140

18 3 23 140

18 4 10 138

19 1 0 20

19 4 9 20

20 2 20 49

20 3 16 43

21 2 11 78

21 3 12 85

21 4 29 170

22 2 7 66

22 4 32 127

23 3 12 76

23 4 20 74

24 3 9 55

24 4 3 26

Data structure: list(N=50, NS=24, NT=4, R=structure(.Data=c(1,0.5,0.5,0.5,1,0.5,0.5,0.5,1), .Dim=c(3,3)))

**References**

1. Evans M, Hastings N, Peacock B. Statistical Distributions. New York: Wiley Interscience; 1993.

2. Armitage P, Berry G. Statistical Methods in Medical Research. Oxford: Blackwell Science Limited; 2001.

3. Lu G, Ades AE. Modeling between-trial variance structure in mixed treatment comparisons. *Biostatistics* 2009; **10**: 792-805.

4. Alvarez I, Niemi J, Simpson M. Bayesian inference for a covariance matrix. *arXiv* 2014: 1408.4050v2.

5. Rao CR. Linear Statistical Inference and its Applications. Hoboken, NJ, USA: John Wiley & Sons, Inc.; 1973.

6. Dias S, Welton NJ, Sutton AJ, Ades AE. NICE DSU Technical Support Document 2: A Generalised Linear Modelling Framework for Pairwise and Network Meta-Analysis of Randomised Controlled Trials. *National Institute for Health and Clinical Excellence* 2011.

7. Turner RM, Jackson D, Wei Y, Thompson SG, Higgins JPT. Predictive distributions for between-study heterogeneity and simple methods for their application in Bayesian meta-analysis. *Statistics in Medicine* 2015; **34**(6): 984-98.

| **Table S1** Predictive distributions^1^ for between-study heterogeneity variance, across 80 different settings (extracted directly from Turner et al.^7^). | | | | | |
| --- | --- | --- | --- | --- | --- |
| **Outcome type** | **Intervention comparison type** | | | | |
|  | Pharmacological vs. Placebo/Control | Pharmacological vs. Pharmacological | Non-pharmacological† vs. Placebo/Control | Non-pharmacological† vs. Pharmacological | Non-pharma.† vs.  Non-pharma.† |
| All-cause mortality | LN(-3.95,1.34^2^) | LN(-4.18,1.41^2^) | LN(-4.17,1.55^2^) | LN(-2.92,1.02^2^) | LN(-3.50,1.26^2^) |
| Obstetric outcomes | LN(-3.52,1.74^2^) | LN(-3.75,1.79^2^) | LN(-3.74,1.91^2^) | LN(-2.49,1.50^2^) | LN(-3.08,1.68^2^) |
| Cause-specific mortality/major morbidity event/composite (mortality or morbidity) | LN(-3.71,1.74^2^) | LN(-3.95,1.79^2^) | LN(-3.93,1.91^2^) | LN(-2.68,1.51^2^) | LN(-3.27,1.68^2^) |
| Resource use/hospital stay/process | LN(-2.34,1.74^2^) | LN(-2.58,1.79^2^) | LN(-2.56,1.91^2^) | LN(-1.31,1.50^2^) | LN(-1.90,1.68^2^) |
| Surgical/device related success/failure | LN(-2.14,1.74^2^) | LN(-2.37,1.79^2^) | LN(-2.36,1.91^2^) | LN(-1.11,1.50^2^) | LN(-1.69,1.68^2^) |
| Withdrawals/drop-outs | LN(-2.99,1.74^2^) | LN(-3.23,1.79^2^) | LN(-3.21,1.91^2^) | LN(-1.96,1.51^2^) | LN(-2.55,1.68^2^) |
| Internal/external structure-related outcomes | LN(-2.71,1.74^2^) | LN(-2.94,1.79^2^) | LN(-2.93,1.92^2^) | LN(-1.67,1.51^2^) | LN(-2.26,1.68^2^) |
| General physical health indicators | LN(-2.29,1.53^2^) | LN(-2.53,1.58^2^) | LN(-2.51,1.72^2^) | LN(-1.26,1.25^2^) | LN(-1.85,1.46^2^) |
| Adverse events | LN(-1.87,1.52^2^) | LN(-2.10,1.58^2^) | LN(-2.10,1.71^2^) | LN(-0.84,1.24^2^) | LN(-1.43,1.45^2^) |
| Infection/onset of new disease | LN(-2.49,1.52^2^) | LN(-2.73,1.58^2^) | LN(-2.71,1.71^2^) | LN(-1.46,1.24^2^) | LN(-2.05,1.45^2^) |
| Signs/symptoms reflecting continuation/end of condition | LN(-2.06,1.51^2^) | LN(-2.29,1.58^2^) | LN(-2.28,1.71^2^) | LN(-1.03,1.24^2^) | LN(-1.61,1.45^2^) |
| Pain | LN(-1.83,1.52^2^) | LN(-2.06,1.58^2^) | LN(-2.05,1.71^2^) | LN(-0.80,1.25^2^) | LN(-1.38,1.45^2^) |
| Quality of life/functioning (dichotomised) | LN(-2.54,1.54^2^) | LN(-2.78,1.60^2^) | LN(-2.77,1.73^2^) | LN(-1.51,1.27^2^) | LN(-2.10,1.47^2^) |
| Mental health indicators | LN(-2.12,1.53^2^) | LN(-2.35,1.60^2^) | LN(-2.34,1.72^2^) | LN(-1.09,1.27^2^) | LN(-1.67,1.47^2^) |
| Biological markers (dichotomised) | LN(-1.77,1.52^2^) | LN(-2.00,1.58^2^) | LN(-1.99,1.71^2^) | LN(-0.74,1.24^2^) | LN(-1.33,1.45^2^) |
| Subjective outcomes (various)† | LN(-2.70,1.52^2^) | LN(-2.93,1.58^2^) | LN(-2.92,1.71^2^) | LN(-1.67,1.25^2^) | LN(-2.26,1.45^2^) |

^1^Fitted distributions reported as log-Normal($\mu$,$\sigma^{2}$), where *μ* and *σ* are the mean and SD on the log scale.

|  | |  | |  | |
| --- | --- | --- | --- | --- | --- |
| **Table S2** Comparison of eight treatments^1^ for localised prostate cancer when assuming equal, proportional or unequal heterogeneity variances: posterior medians and 95% credible intervals for between-trial standard deviations. | | | | | | | | | | |
|  | Approach 1:  Equal variances  (vague prior) | | Approach 1:  Equal variances  (inf. prior) | | Approach 2:  Proportional variances  (vague priors) | | Approach 2:  Proportional variances  (inf. priors) | Approach 3:  Unequal variances  (vague priors) | Approach 3:  Unequal variances  (inf. priors) | Approach 4:  Scaled inverse Wishart  (inf. prior) |
| $\tau_{BC}$ | - | | - | | 0.09 (0.003,0.36) | | 0.12 (0.04,0.30) | 0.61 (0.10,1.7) | 0.11 (0.03,0.36) | 0.08 (0.03,0.30) |
| $\tau_{BD}$ | - | | - | | 0.09 (0.003,0.36) | | 0.12 (0.04,0.30) | 0.81 (0.13,1.85) | 0.11 (0.03,0.40) | 0.08 (0.03,0.31) |
| $\tau_{BE}$ | - | | - | | 0.09 (0.003,0.36) | | 0.12 (0.04,0.30) | 0.56 (0.09,1.67) | 0.11 (0.03,0.36) | 0.08 (0.03,0.30) |
| $\tau_{BF}$ | - | | - | | 0.09 (0.003,0.36) | | 0.12 (0.04,0.30) | 0.57 (0.10,1.68) | 0.11 (0.03,0.36) | 0.08 (0.03,0.30) |
| $\tau_{BG}$ | - | | - | | 0.09 (0.003,0.36) | | 0.12 (0.04,0.30) | 0.93 (0.16,1.89) | 0.12 (0.03,0.45) | 0.08 (0.03,0.32) |
| $\tau_{BH}$ | - | | - | | 0.09 (0.003,0.36) | | 0.12 (0.04,0.30) | 0.87 (0.15,1.87) | 0.11 (0.03,0.44) | 0.08 (0.03,0.31) |
| $\tau_{CD}$ | - | | - | | 0.09 (0.003,0.36) | | 0.12 (0.04,0.30) | 0.70 (0.11,1.81) | 0.11 (0.03,0.38) | 0.08 (0.03,0.29) |
| $\tau_{CE}$ | - | | - | | 0.09 (0.003,0.36) | | 0.12 (0.04,0.30) | 0.50 (0.09,1.58) | 0.10 (0.03,0.34) | 0.08 (0.03,0.29) |
| $\tau_{CF}$ | - | | - | | 0.09 (0.003,0.36) | | 0.12 (0.04,0.30) | 0.52 (0.09,1.58) | 0.11 (0.03,0.34) | 0.08 (0.03,0.29) |
| $\tau_{CG}$ | - | | - | | 0.09 (0.003,0.36) | | 0.12 (0.04,0.30) | 0.90 (0.15,1.87) | 0.11 (0.03,0.44) | 0.08 (0.03,0.31) |
| $\tau_{CH}$ | - | | - | | 0.09 (0.003,0.36) | | 0.12 (0.04,0.30) | 0.81 (0.13,1.84) | 0.11 (0.03,0.43) | 0.08 (0.03,0.31) |
| $\tau_{DE}$ | - | | - | | 0.09 (0.003,0.36) | | 0.12 (0.04,0.30) | 0.72 (0.11,1.81) | 0.11 (0.03,0.38) | 0.08 (0.03,0.30) |
| $\tau_{DF}$ | - | | - | | 0.09 (0.003,0.36) | | 0.12 (0.04,0.30) | 0.72 (0.11,1.82) | 0.11 (0.03,0.38) | 0.08 (0.03,0.31) |
| $\tau_{DG}$ | - | | - | | 0.09 (0.003,0.36) | | 0.12 (0.04,0.30) | 1.04 (0.18,1.95) | 0.12 (0.03,0.48) | 0.08 (0.03,0.32) |
| $\tau_{DH}$ | - | | - | | 0.09 (0.003,0.36) | | 0.12 (0.04,0.30) | 0.99 (0.17,1.93) | 0.12 (0.03,0.47) | 0.08 (0.03,0.32) |
| $\tau_{EF}$ | - | | - | | 0.09 (0.003,0.36) | | 0.12 (0.04,0.30) | 0.37 (0.07,1.33) | 0.10 (0.03,0.33) | 0.08 (0.03,0.26) |
| $\tau_{EG}$ | - | | - | | 0.09 (0.003,0.36) | | 0.12 (0.04,0.30) | 0.86 (0.13,1.86) | 0.11 (0.03,0.44) | 0.08 (0.03,0.31) |
| $\tau_{EH}$ | - | | - | | 0.09 (0.003,0.36) | | 0.12 (0.04,0.30) | 0.79 (0.13,1.84) | 0.11 (0.03,0.42) | 0.08 (0.03,0.31) |
| $\tau_{FG}$ | - | | - | | 0.09 (0.003,0.36) | | 0.12 (0.04,0.30) | 0.86 (0.13,1.85) | 0.11 (0.03,0.44) | 0.08 (0.03,0.31) |
| $\tau_{FH}$ | - | | - | | 0.09 (0.003,0.36) | | 0.12 (0.04,0.30) | 0.8 (0.13,1.84) | 0.11 (0.03,0.43) | 0.08 (0.03,0.31) |
| $\tau_{GH}$ | - | | - | | 0.09 (0.003,0.36) | | 0.12 (0.04,0.30) | 1.08 (0.2,1.98) | 0.12 (0.03,0.51) | 0.08 (0.03,0.33) |

^1^ Observational management (A); Prostatectomy (B); Conventional RT (C); Conventional RT hypofractionated (D); Conformal low dose RT (E); Conformal high dose RT (F); Conformal low dose RT hypofractionated (G); Cryotherapy (H)
